# A single dose polyanhydride-based nanovaccine against paratuberculosis infection

**DOI:** 10.1038/s41541-020-0164-y

**Published:** 2020-02-14

**Authors:** Akanksha Thukral, Kathleen Ross, Chungyi Hansen, Yashdeep Phanse, Balaji Narasimhan, Howard Steinberg, Adel M. Talaat

**Affiliations:** 1 Department of Pathobiological Sciences, University of Wisconsin, Madison, WI 53706 USA; 2 Department of Chemical and Biological Engineering, Iowa State University, Ames, IA 50010 USA; 3 Nanovaccine Institute, Iowa State University, Ames, IA 50010 USA; 4 Pan Genome Systems, INC., Madison, WI 53719 USA

**Keywords:** Adjuvants, Vaccines, Immunology

## Abstract

*Mycobacterium avium* subsp*. paratuberculosis* (*M. paratuberculosis*) causes Johne’s disease in ruminants and is characterized by chronic gastroenteritis leading to heavy economic losses to the dairy industry worldwide. The currently available vaccine (inactivated bacterin in oil base) is not effective in preventing pathogen shedding and is rarely used to control Johne’s disease in dairy herds. To develop a better vaccine that can prevent the spread of Johne’s disease, we utilized polyanhydride nanoparticles (PAN) to encapsulate mycobacterial antigens composed of whole cell lysate (PAN-Lysate) and culture filtrate (PAN-Cf) of *M. paratuberculosis*. These nanoparticle-based vaccines (i.e., nanovaccines) were well tolerated in mice causing no inflammatory lesions at the site of injection. Immunological assays demonstrated a substantial increase in the levels of antigen-specific T cell responses post-vaccination in the PAN-Cf vaccinated group as indicated by high percentages of triple cytokine (IFN-γ, IL-2, TNF-α) producing CD8^+^ T cells. Following challenge, animals vaccinated with PAN-Cf continued to produce significant levels of double (IFN-γ, TNF-α) and single cytokine (IFN-γ) secreting CD8^+^ T cells compared with animals vaccinated with an inactivated vaccine. A significant reduction in bacterial load was observed in multiple organs of animals vaccinated with PAN-Cf, which is a clear indication of protection. Overall, the use of polyanhydride nanovaccines resulted in development of protective and sustained immunity against Johne’s disease, an approach that could be applied to counter other intracellular pathogens.

## Introduction

*M. paratuberculosis* is the causative pathogen of Johne’s disease (JD) characterized by chronic gastroenteritis, diarrhea, weight loss and low milk yield in ruminants.^1^ While JD is a worldwide problem, its prevalence in the United States is estimated to be >90% in dairy herds,^2^ causing a combined loss of $200–250 million to the US dairy industry.^3,4^ The financial losses are incurred due to premature culling of infected animals, decreased milk production, and increased somatic cell infiltration in milk.^5,6^ JD is a slowly progressing disease and can infect 38–40% of the herd before becoming symptomatic in a single animal.^7,8^ Currently there is no treatment for JD and controlling the disease progression by culling the infected animals is very expensive, whereas vaccination offers a reasonable alternative.^9^ Mycopar® (Boehringer Ingelheim) is an oil suspended, heat killed, whole cell vaccine licensed in the United States. However, Mycopar® fails to completely protect against JD^10,11^ and can cause severe inflammatory lesions at the site of injection.^12^ It also poses a health risk to vaccinators due to accidental inoculation, which leads to a chronic inflammatory reaction that potentially requires surgical intervention.^13^ Given the challenges to control JD with the current vaccine, we directed our efforts to develop a more effective and safe vaccine against JD using polyanhydride nanoparticles (PAN).

An ideal vaccine should elicit a robust immune response without causing untoward reactions in the vaccinee or risk to the vaccinator. Another important aspect of vaccine development against Mycobacterial infection is its capability to elicit a polyfunctional T cell response with simultaneous production of pro-inflammatory cytokines by T cells.^14,15^ To elicit robust immunity, antigens are often formulated with adjuvants to prolong their release and enhance their protective immunity. In this study, we used whole cell lysate and culture filtrate proteins encapsulated in biodegradable polyanhydride nanoparticles (adjuvant) that provide sustained release of *M. paratuberculosis* antigens by surface erosion.^16^ PAN-based vaccines (i.e., nanovaccines) have been shown to impart long lasting protective immunity against several infectious diseases including influenza, pneumonic plague, respiratory syncytial virus, and pneumonia, using pathogen-specific protein antigens.^16–22^

The amphiphilicity of the PAN chemistry provides antigen stability and the copolymer composition enables sustained release of the encapsulated immunogens.^21,23–27^ The small size (~200 nm) and large surface area of the nanoparticles allows them to carry antigens across cellular membranes and deliver them to their targets.^28–30^ In addition, their molecular chemistry and size has pathogen-mimicking characteristics, allowing PAN to be engulfed by, persist within, and subsequently stimulate antigen presenting cells (APCs).^31,32^ Polyanhydride particles on their own exhibit adjuvant-like properties by activating APCs^31–33^ and inducing both humoral and cell-mediated immune responses;^17,33–35^ formulating them with immune-stimulatory antigens results in protective immunity.^22,35^ Finally, these particles have been shown to be safe and induce less inflammation at the administration site compared with traditional adjuvants such as Alum and incomplete Freund’s adjuvant.^36,37^

**Fig. 1:**
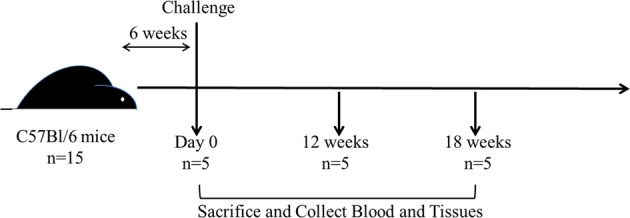
Experimental design for vaccination and challenge. Five- to eight-week-old female C57BL/6 mice were vaccinated with subcutaneous injection and challenged six weeks later with virulent strain of *M. paratuberculosis* JTC-1285 by the intraperitoneal route. Mice (*n* = 5/group) were sacrificed at various time points. Tissues and blood samples were collected to measure bacterial burden, cytokine levels and histopathology.

*M. paratuberculosis* whole cell lysate and culture filtrate proteins have been shown to exhibit immunogenic properties and have previously been evaluated as a potential vaccine.^38,39^ Therefore, we utilized *M. paratuberculosis* antigens together with PAN to formulate nanovaccines that can elicit robust and sustainable protective immune responses. In this study, a single, subcutaneous dose of nanovaccine in C57BL/6 mice was evaluated for protection against *M. paratuberculosis* JTC-1285 challenge in comparison to both inactivated and live vaccine candidates. The live vaccine candidate *lip*N, developed previously by our group, was constructed by knockout of a fatty acid lipase/esterase gene *lip*N from *M. paratuberculosis* K10. This gene was significantly upregulated in *M. paratuberculosis* shed in the cow feces, as revealed by transcriptional profiling.^40^ Also, *lipN* mutant was analyzed and found to be attenuated in mice as indicated by reduced histopathological lesions and colonization of the liver.^41^ Its protective efficacy has been observed in goats challenged by virulent *M. paratuberculosis* strain.^42^ The study was conducted in two phases, viz: Trial I and Trial II. In the trial I studies, the focus was on the safety of the nanovaccine formulations while in the trial II studies, the focus was on the efficacy of nanovaccine formulations (Fig. 1).

## Results

### Nanovaccine characterization and safety

**Fig. 2:**
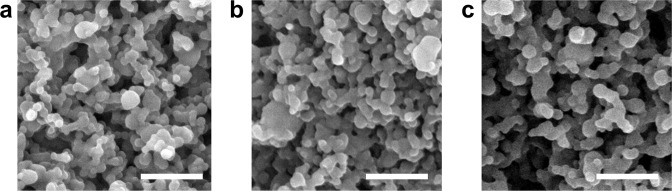
Characterization of *M. paratuberculosis* antigen-containing nanoparticle-based vaccines. Scanning electron microscopy images of 20:80 CPTEG:CPH nanoparticles loaded with 2.5% whole cell lysate (**a**) and 2.5% culture filtrate (**b**) showed similar spherical morphology and size (~200 nm) as blank nanoparticles (**c**). Scale bar = 1 μm.

Scanning electron photomicrographs of *M. paratuberculosis* lysate-encapsulated (PAN-Lysate) and culture-filtrate (PAN-Cf)-encapsulated polyanhydride nanoparticles showed similar spherical morphology and size as blank (i.e., empty) nanoparticles, indicating that antigen encapsulation did not change the average diameter, which was ca. 200 nm (Fig. 2). The encapsulation efficiency of the lysate into the nanoparticles was 40.0 ± 1.9% and that of the culture filtrate was 26.0 ± 0.4% and 2.5 wt% of the protein content of the lysate or culture filtrate (Cf) was encapsulated into the particles. To evaluate nanovaccine safety, we monitored immunized mice on a daily basis. Animals vaccinated with Mycopar® gradually developed an abscess at the injection site which progressed and persisted throughout the study (Supplemental Fig. 7). On the other hand, no lesions were observed in nanovaccine-immunized and live attenuated *(lipN)* vaccine immunized animals. At 6 weeks post-vaccination (WPV) and before any challenge, histopathology of vaccinated mice groups demonstrated lymphoid depletion in the spleens of animals immunized with the commercial vaccine, while minimal to moderate lymphocytic infiltration and granulomatous inflammation was observed in the livers of the rest of vaccinated animals regardless of formulation, which is indicative of induced immunity. No pathology was observed in the negative control group (PBS-vaccinated mice).

### Pre-challenge immune responses

**Fig. 3:**
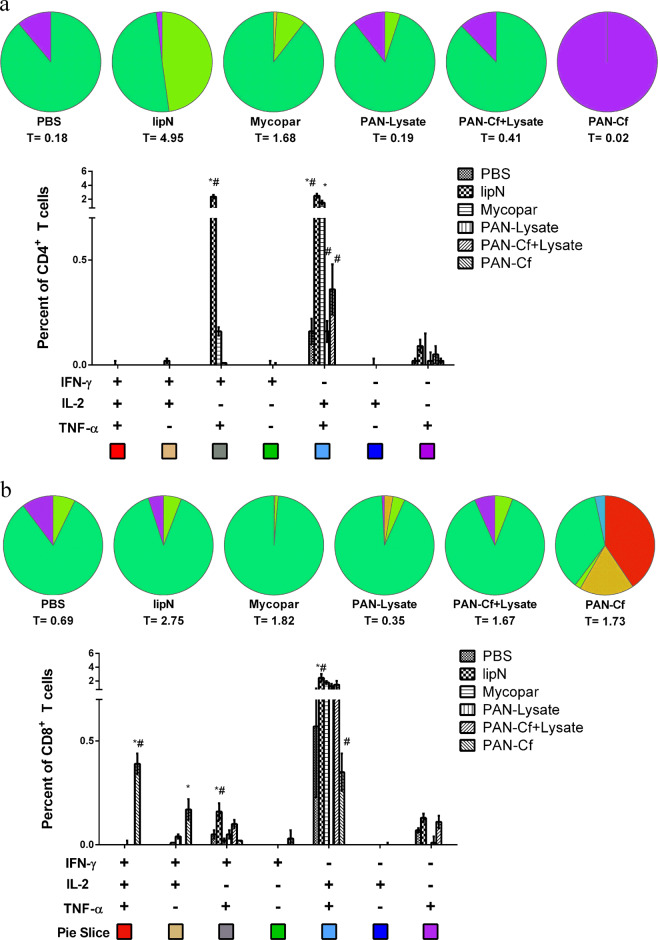
Pre-challenge immune response specific to lysate of *M. paratuberculosis*. C57BL/6 mice (*n* = 5) were immunized with various vaccine groups and 6 WPV, five mice from each group were euthanized. Spleens were harvested; lymphocytes were isolated and stimulated with the *M. paratuberculosis* lysate for 24 h. Cells were then stained for CD4^+^ (**a**) and CD8^+^ (**b**) cell surface markers and intracellular cytokines. The total percentage of T cells secreting particular cytokines are indicated below each pie chart (denoted by T = number). The error bars show the standard error of the mean for five individually analyzed mice. * indicates *p* < 0.05; ** indicates *p* < 0.001. * denotes comparison with PBS while ^#^ denotes comparison with Mycopar®. Results were expressed as the increase in the percentage of the cells with positive staining relative to that of an unstimulated sample stained with the same antibody.

For Trial I (i.e., safety study), the T cell response was evaluated at 6 weeks post-vaccination by performing IFN-γ ELISA on spleen derived lymphocytes (described in Methods) (Supplemental Fig. 1). The Mycopar-vaccinated animals showed significantly higher IFN-γ levels than the rest of the groups (***p < 0.001) (Supplemental Fig. 2a). For Trial II (i.e., efficacy study), spleen derived lymphocytes were stained with various antibody markers and analyzed using flow cytometry. We assessed antigen specific, polyfunctional T cell responses by multi-parametric flow-cytometry. In this analysis, *LipN* vaccinated mice showed significantly higher percentage of double cytokine (IFN-γ,TNF-α) and single cytokine (IFN-γ) producing CD4^+^ T cells as well CD8^+^ T cells in comparison with both PBS and Mycopar® vaccinated animals (Fig. 3a, b). Interestingly, the percentage of triple cytokine producing (IFN-γ, IL-2, TNF-α) CD8^+^ T cells was significantly higher in mice immunized with PAN-Cf when compared with PBS and Mycopar®. Also, the PAN-Cf vaccinated animals exhibited significantly higher double (IFN-γ, IL-2) cytokine secreting CD8^+^ T cells in comparison with PBS vaccinated mice (Fig. 3b). Of note was the breadth of the polyfunctional CD8^+^ T cell response observed from mice immunized with PAN-Cf. In contrast to all the other treatment groups where the majority of the CD8^+^ T cells was dominated by IFN-γ secreting single positive cells, PAN-Cf vaccinated mice showed a broader profile of triple, double and single cytokine secreting CD8^+^ T cells (Fig. 3b).

### Post-challenge immune responses

**Fig. 4:**
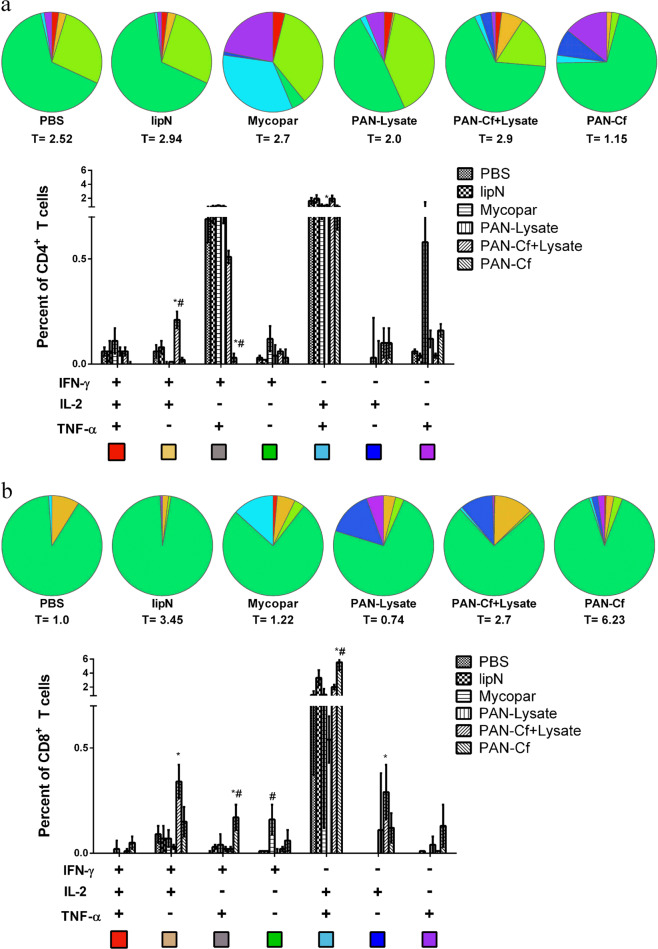
Early cellular responses in vaccine groups following challenge with a wild type strain of *M. paratuberculosis*. Six to eight week-old C57BL/6 mice were immunized with various vaccine candidates. At 6 WPV they were challenged with *M. paratuberculosis* JTC-1285 and euthanized 12 weeks later (12 WPC). The lymphocytes were isolated from the spleens and stimulated with whole cell lysate of *M. paratuberculosis* for 24 h. Cells were then stained for CD4^+^ (**a**) and CD8^+^ (**b**) cell surface markers and intracellular cytokines and were measured by flow cytometry. The total percentage of T cells secreting particular cytokines are indicated below each pie chart (denoted by T = number). The error bars show the standard error of the mean for five individually analyzed mice. * indicates *p* < 0.05; ** indicates *p* < 0.001. * denotes comparison with PBS while ^#^ denotes comparison with Mycopar®. Results were expressed as the increase in the percentage of the cells with positive staining relative to that of an unstimulated sample stained with the same antibody.

To evaluate T cell response in Trial I/safety study IFN-γ ELISA was performed and result of which depicted no significant differences in IFN-γ levels among the groups (Supplemental Fig. 2b) while at 12 weeks post challenge Mycopar and PAN-lysate vaccinated animals showed significantly higher IFN-γ levels as compared with control animals given PBS (Supplemental Fig. 2c). T cell responses for the Trial II/Vaccine efficacy study were evaluated by flow cytometry for which spleens from vaccinated mice were collected at 12 and 18 weeks post-challenge (WPC). At 12 WPC, multi-parametric flow cytometry analysis indicated that mice immunized with PAN-Cf elicited a significantly higher percent of antigen specific double cytokine (IFN-γ, TNF-α^+^) and single cytokine (IFN-γ) producing CD8^+^ T cells compared with non-vaccinated and Mycopar® vaccinated mice (Fig. 4). In addition, PAN-Cf and Mycopar®-vaccinated animals also displayed low levels of triple cytokine secreting CD8^+^ T cells. Similar to the pre-challenge CD8^+^ T immune response, mice immunized with PAN-Cf showed a broader profile of cytokine secreting cells at 12 WPC (see pie chart in Fig. 3). The cumulative percentage of CD8^+^ T cells that were either triple, double or single cytokine secretors was also higher in animals vaccinated with PAN-Cf (total percentage of cells secreting cytokine; T = 6.23) in contrast to that in animals vaccinated with the other formulations, indicating the robustness of the induced CD8^+^ T cell response. Also, animals receiving PAN-Cf + Lysate showed significantly higher levels of double cytokine secreting (IFN-γ, IL-2) CD8^+^ T cells in comparison with animals that received PBS and significantly higher levels of double cytokine secreting (IFN-γ, IL-2) CD4^+^ T cells in comparison with animals receiving both PBS and Mycopar®. At 18 WPC, the percentages of mycobacterial antigen-specific double positive CD8^+^ T and CD4^+^ T cells (IFN-γ^+^IL-2^+^) were significantly higher in PAN-Cf vaccinated mice compared with PBS-vaccinated mice (Supplemental Fig. 3).

### Protection against challenge with virulent strains of *M. paratuberculosis*

In spite of the fact that safety was the main goal of the Trial I studies, we were able to evaluate the protective efficacy of each vaccine candidate at 6WPC when bacterial colonization remained similar in organs of all vaccinated groups (Supplemental Fig. 4). At 12WPC, the bacterial load was significantly lower in the spleens and mesenteric lymph nodes of Mycopar and PAN-Lysate and Lysate vaccinated groups in comparison with non-vaccinated group. Livers of Mycopar and PAN-Lysate vaccine group showed significant reduction in comparison with PBS group (Supplemental Fig. 5).

**Fig. 5:**
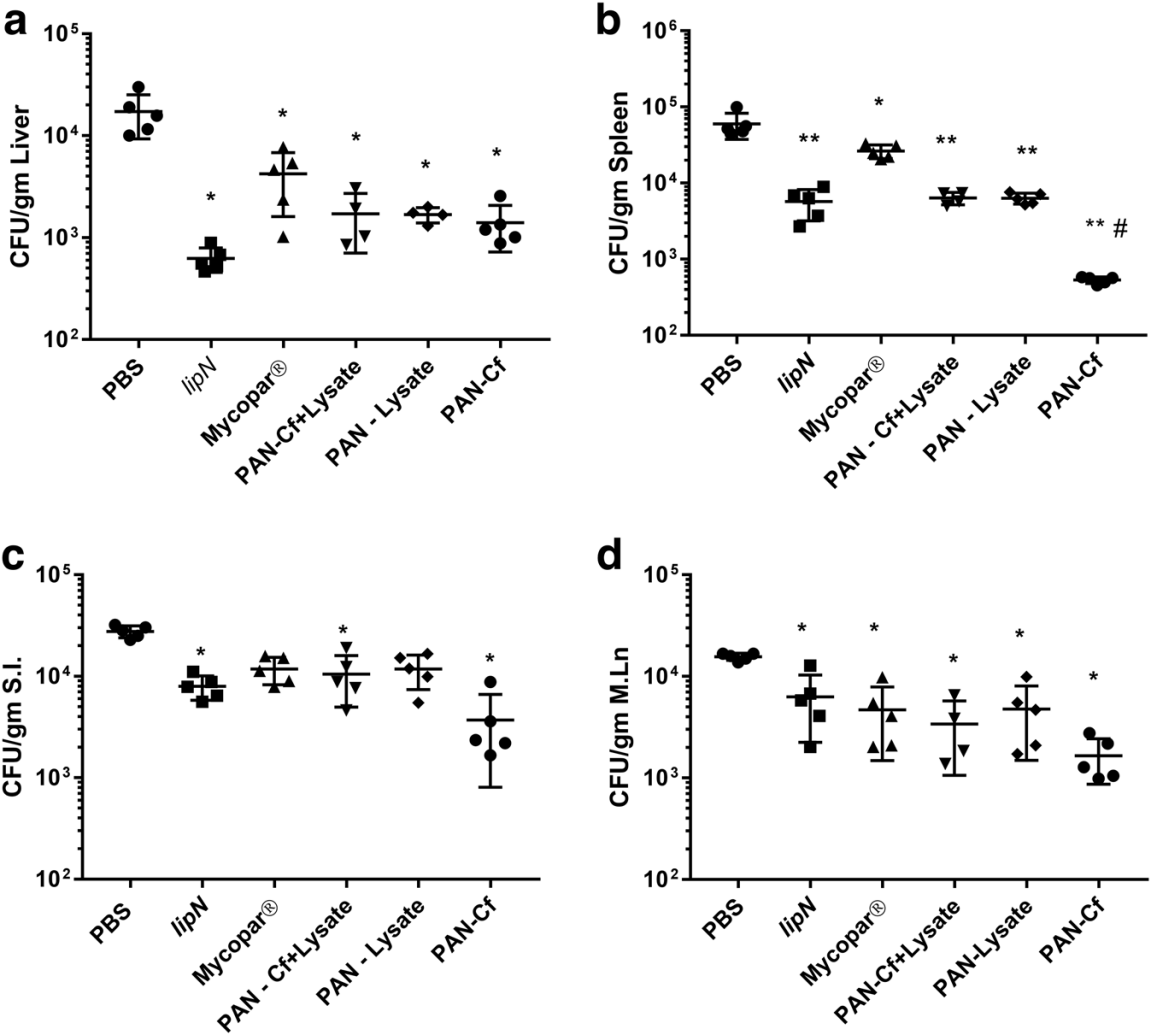
Protection against challenge strain of *M. paratuberculosis*. Levels of *M. paratuberculosis* colonization in body organs of mice at 12 WPC. The total colony counts for each individual animal for each vaccine groups are shown in spleen (**a**), liver (**b**), small intestine (**c**) and mesenteric lymph node (**d**). Error bars indicate standard deviation. * indicates *p* < 0.05; ** indicates *p* < 0.001. * denotes comparison with PBS while ^#^ denotes comparison with Mycopar®.

To better evaluate protection offered by each vaccine formulation in Trial II/Efficacy study against recent isolates of *M. paratuberculosis*, we quantified the level of bacterial tissue colonization following a challenge with *M. paratuberculosis* JTC1285, a clinical isolate of the bovine origin.^43^ As expected, mice that received PBS had high levels of bacterial load in all the tissues studied (liver, spleen, intestine and mesenteric lymph node) at 12 WPC (Fig. 5). All the vaccinated mice showed significantly lower bacterial burden in the liver in comparison with PBS-treated mice (Fig. 5a). Bacterial load was significantly lower in the spleens of all vaccine groups (including Mycopar®, *lipN*, and PAN-Lysate) with a two-log reduction observed in the spleens of animals vaccinated with PAN-Cf in comparison with the load in the spleens of animals that received PBS. The PAN-Cf immunized mice also showed significant reduction in bacterial load burden compared with Mycopar® vaccinated mice (Fig. 5b). Interestingly, Mycopar® did not provide any protection in terms of a reduced bacterial load in the small intestine (Fig. 5c) compared with the PBS-treated animals. In contrast, mice vaccinated with *lipN* mutant, PAN-Cf + Lysate and PAN-Cf had significantly lower mycobacterial colonization levels in the small intestine. All mouse groups displayed a reduction in bacterial colonization of the mesenteric lymph nodes compared with the PBS control (Fig. 5c, d). At 18 WPC, no significant differences were observed in the bacterial colonization in the mouse tissues among any of the vaccine groups, including PBS (Supplemental Fig. 6).

### Histopathology

**Fig. 6:**
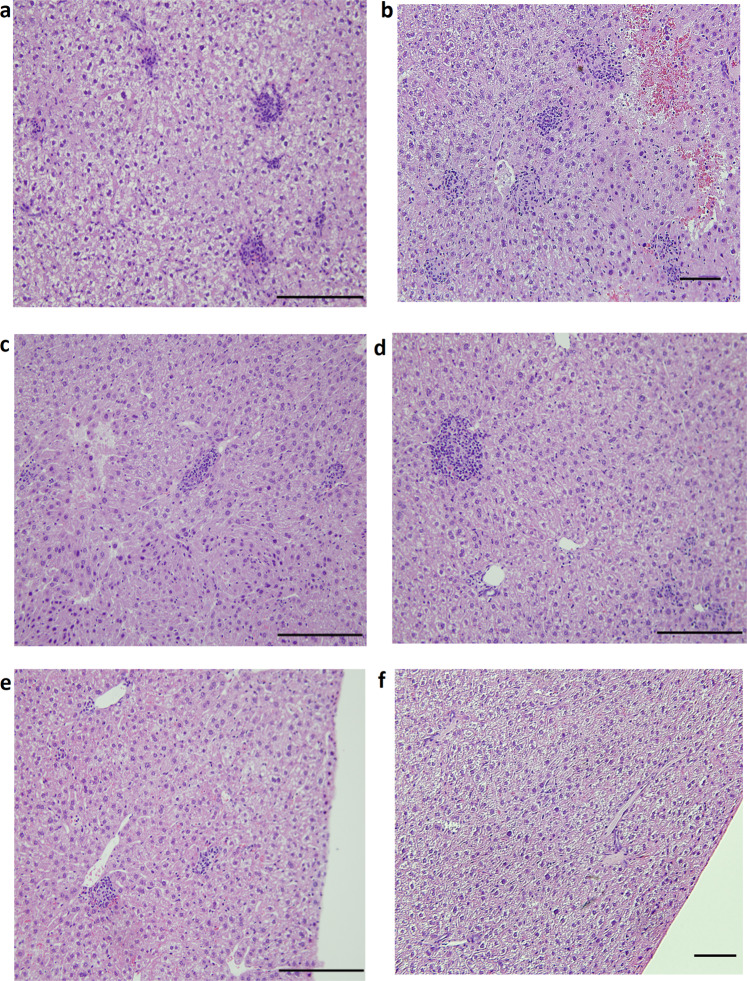
Histopathology of vaccinated and challenged mice. Liver tissues from PBS (**a**), Mycopar® (**b**), *lipN* (**c**), PAN-Lysate (**d**), PAN-Cf + Lysate (**e**), PAN-Cf (**f**) vaccinated mice were harvested at 12 WPC. Tissues were sectioned to 5 µm slices and stained with H&E and analyzed at 20× magnification. Scale bar: 100 µm.

To analyze the level of tissue damage induced by challenge with the wild type *M. paratuberculosis* JTC1285 strain, we performed histopathology of the main body organs in all vaccine groups. At 12 WPC, all animals administered PBS had granulomatous inflammation in the liver while only 40% of the animals receiving the PAN-Lysate, PAN-Cf + Lysate, or *lipN* vaccine exhibited minimal to mild pathology (Table 1; Fig. 6). The granulomatous lesions in livers involved variable size aggregates of lymphocytes with macrophages visible in some lesions. In the Mycopar® vaccinated group, 60% of the animals had minimal to moderate granulomatous inflammation in the liver (Table 1). At 18 WPC, granulomatous and lymphocytic inflammation were larger in size and involved more sections of the liver in all groups with no significant differences among vaccine groups.

**Table 1:** Histopathology scores of liver at 12 WPC.

Granulomatous inflammation (GI) in liver						
Vaccine groups	Severity score^a^					% of animals with GI
PBS	2	3	1	1	1	100
Mycopar®	2	N	N	3	1	60
PAN-Lysate	2	N	N	1	N	40
PAN-Cf + Lysate	2	N	N	1	N	40
PAN-Cf	1	1	1	1	N	80
*lipN*	1	N	N	2	N	40

^a^Severity index of granulomatous lesions for individual animals, as follows: N = normal, 1 = minimal, 2 = mild, 3 = moderate, 4 = severe, 5 = massive.

## Discussion

Despite many challenges and shortcomings, vaccination against MAP is still considered to be the most efective strategy to curb Johne’s disease.^44^ The commercially available vaccines, such as Mycopar®, Gudair®, and Silirum®, are comprised of whole inactivated MAP and provide moderate protection at best.^45,46^ The limited benefits provided by these vaccines are overshadowed by their drawbacks, which include granulomatous reaction at the injection site and poor protection against bacterial tissue colonization and shedding.^46,47^ In this report, we utilized the polyanhydride nanovaccine platform technology to design a safer and more efficacious vaccine aginst JD. Previous studies have shown that PANs can encapuslate a diverse array of biologics including subunit proteins, peptides, and antibiotics.^17,35^ Here we demonstrate that polyanhydride nanoparticles can encapsulate complex payloads such as *M. paratuberculosis* proteins (from whole cell lysates and culture filtrate) and induce effective immune responses in mice indicating that the immunogenicity of the encapsulated cargo is intact. This novel delivery approach to inactivated vaccine improve their efficacy but maintain their safety profile. The strategy of using *M. paratuberculosis* proteins in both encapsulated and soluble form enables primed (provided by the soluble protein) and sustained (provided by the nanoparticle-encapsulated protein) immune responses.^16,33,48^ Consistent with previous studies that examined the safety of inactivated vaccines (e.g., Mycopar®),^47,49^ we observed abscess-like lesions at the inoculation site in several mice vaccinated with Mycopar® (Supplemental Fig. 7). In contrast, no adverse injection site reactions were observed in PAN-vaccinated mice, clearly indicating their excellent safety profile. These results add to the body of literature on the minimal reacotogenicity of polyahydride nanovaccines, as demonstrated previously.^36,50^

Although oral infection and mucosal immunizations against *M. paratuberculosis* could be more benificial in the target host (cattle),^51^ we selected parental infection and subcutaenous injection of mice to test vaccine formulas using an entery level model for vaccine testing against paratuberculosis.^52^ For both the murine and bovine models of paratuberculosis, CD4^+^ and CD8^+^ effector T cells play a crucial role in eliciting protective cell mediated immunity against mycobacterial infection.^53,54^ Effector T cells producing multiple pro-inflammatory cytokines such as IFN-γ,^55^ TNF-α,^56^ and IL-2^57^ have been shown to be associated with protection against various intracellular pathogens including *M. tuberculosis*.^14,15^ Even though the exact mechanism(s) of polyfunctional T cell mediated protection are still not clear, it has been shown that two or more cytokines can work synergistically to control infection, as in the case of a closely related mycobacterium, *M. tuberculosis*^58^ and *Leishmania* spp.^59^ Indeed, PAN-Cf vaccinated (prechallenged) mice exhibited polyfunctional CD8^+^ T cells (IFN-γ+, IL-2+, TNFα+) but showed superior protection (significantly lower bacterial burden) compared with both non-vaccinated (all organs) and Mycopar (only spleen) vaccinated groups. We hypothesize that protection can be attributed to a robust polyfunctional CD8^+^ T cell response, especially for the nanovaccine formulas. Recently, nano-peptide based adjuvant enhanced BCG primed immune response by induction of a robust polyfunctional CD8^+^ T cells.^60^ Overall, the presented analyses (Figs 3, 4) clearly indicated robust induction of polyfunctional T cell responses in mice immunized by the nanovaccines as depicted by higher triple and double cytokine producing CD8^+^ T cells when compared with non-vaccinated (PBS) and Mycopar®-vaccinated mice. It is noteworthy to mention here that for *M. tuberculosis* vaccines, polyfunctional T cells were mainly CD4^+^ unlike the predominantly CD8^+^ population observed in animals vaccinated with the PAN-Cf group. This is likely induced by the inclusion of polyanhydride nanoparticles in the PAN-Cf group, consistent with previous observations.^61,62^

The nanoparticle chemistry used in this study (i.e., 20:80 CPTEG:CPH) was rationally selected based on previous reports showing its potency as an adjuvant as exhibited by robust induction of cellular immune responses.^35^ Similarly, in this study PAN-Cf vaccinated mice at pre-challenge not only induced a polyfunctional CD8^+^ T cell response but also had more breadth as indicated by induction of more types of cytokine secreting cells. The robust polyfunctional T cell response observed in PAN-Cf vaccinated animals for 12 WPC may be attributed to sustained release of antigen from polyanhydride particles.^29^ Remarkably, PAN-Cf^+^ Lysate was able to produce double cytokine (IFN-γ^+^IL-2^+^) CD4^+^ T cells significantly higher than PBS and Mycopar® and exhibited lower bacterial burden in all the mice tissues as compared with non-vaccinated mice (Fig. 6). Most importantly, PAN-Cf vaccinated mice had the lowest bacterial burden in three out of the four tissues evaluated. Moreover, despite this significant reduction was not maintained at 18 WPC, the level of polyfunctional T cells remained robust at this prolonged time. This indicates that several other paramters (beyond the scope of present study), such as Th-17 induction, tissue homing properties of T cells, memory and effector phenotype could also play critical roles.^63,64^ These parameters deserve more attention, especially as we advance vaccine testing to ruminant models.

As expected, histological lesions post-challenge correlated with protection based on bacterial burden in body organs. For example, granulomatous lesions that are typical of mycobacterial infection were seen in the liver of PBS and Mycopar®-vaccinated animals in higher frequency than in the *lipN* or PAN-vaccinated animals (Table 1), consistent with the levels of mycobacterial colonization. Similar to other vaccine candidates (*lipN* mutant), PAN-based vaccines were able to significantly lower *M. paratuberculosis* levels in the liver, spleen and lymph nodes but did not prevent dissemination of infection. Approaches focused on developing significant mucosal immunity at the main site of *M. paratuberculosis* entry (intestine) could definitely reduce organ dissemination of the infection. Overall, the nanovaccines and *lipN* mutant were able to impart superior protective imunity against *M. paratuberculosis* challenge in mice compared with a commercial vaccine, Mycopar®. Further, in contrast to Mycopar®, nanovaccines were also well tolerated and did not induce any adverse reactions at the site of injection. Both these features makes PAN nanovaccines ideal for further testing in larger animals such as goats and cattle following mucosal immunization and oral infections, to memic natural infection. Although promising, nanovaccines have room for improvement before becoming suitable for field applications. For example, methods to improve the encapsulation efficiency of complex protein mixtures like whole cell lysate into PAN are highly desirable and if developed, could improve vaccine production and facilitate immunization. Such strategies could open the door for the development of more effective vaccines targeting other intracellular pathogens such as *M. tuberculosis*.

## Methods

### Bacterial strains and growth conditions

For safety experiments, *M. paratuberculosis* -K10 was used to prepare lysate while for efficacy experiments, *M. paratuberculosis* strain JTC-1285 was used to derive lysate and culture-filtrate (Cf) proteins for vaccine formulation. Both isolates belong to C-type of *M. paratuberculosis* with almost identical genomes.^43^ The same strains were used for the animal challenge studies, as described below. The strains were grown in Middlebrooks 7H9 broth (Difco, Sparks, MD) supplemented with 0.5% glycerol (v/v) and 10% (v/v) ADC (albumin, dextrose, catalase) or Tween 80 and 1 mg/mL mycobactin J (Allied Monitor, Fayette, MO) in a shaking incubator at 37 °C until it reached the log phase (OD_600_ = 0.5–1.0).^43^ The *M. paratuberculosis* field isolate, JTC-1285, was sub-cultured in Watson Reid medium,^65,66^ modified by supplementing 1 mg/mL mycobactin J (Allied Monitor, Fayette, MO). The Watson Reid medium enabled culture filtrate proteins to be analyzed free of bovine serum albumin (BSA) contamination. The live attenuated vaccine strain, *M. paratuberculosis ΔlipN* mutant (*lipN* mutant) was constructed by an in-frame deletion of 1.1 kb of the *lipN* gene from *M. avium* subsp. *paratuberculosis* strain K-10 and grown in Middlebrooks 7H9 broth in presence of 30 μg/mL hygromycin.^40^ This vaccine construct was used as a control to evaluate nanovaccine performance.

### Preparation of culture filtrate and lysate protein

*M. paratuberculosis* K10 and JTC-1285 cultures were centrifuged in pre-weighed 50 mL conical tubes at 3200 × *g* for 15 min at room temperature. The supernatant was filter sterilized with 0.22 μm polyethersulfone (PES) filter (Nalgene). Further, it was size fractionated by ultrafiltration (Corning Ultra spin columns, 5000 MWCO) and the filtered volume retained on the membrane was dialyzed twice against 10 mM phosphate buffered saline (PBS) (pH 7.2). The concentrated culture filtrate proteins were quantified using bicinchoninic acid kit (Pierce) and stored at −20 °C. To obtain the lysate, the bacterial cell pellet was resuspended in protein lysis buffer (100 mM Tris–Cl, 100 mM NaCl, 5 mM MgCl_2_, 1 mM PMSF, complete ultra protease inhibitor cocktail, pH 7.5) and placed in microcentrifuge tubes containing 0.1-mm zirconia/glass beads. Tubes were shaken in the Mini Bead-beater cell disrupter for four 45 s pulses followed by 1-min incubation on ice. Cellular debris and beads were pelleted by centrifugation at 3200 × *g* for 20 min. The supernatant was quantified for protein using bicinchoninic acid kit (Thermo Fisher Scientific, Rockford, IL) and stored at −20 °C.

### Nanoparticle formulation and protein encapsulation

Diacids of 1,8-bis(p-carboxyphenoxy)-3,6-dioxaoctane (CPTEG) and 1,6-bis(p-carboxyphenoxy)hexane (CPH) were synthesized as described in detail.^67,68^ Next, melt polycondensation was used to synthesize 20:80 CPTEG:CPH copolymer. The purity and molecular weight of the copolymer were verified using ^1^H nuclear magnetic resonance spectroscopy (VXR 300 MHz, Varian, Palo Alto, CA) before proceeding to nanoparticle synthesis. Nanoparticles were synthesized using solid-oil-oil double emulsion nanoprecipitation.^31^ Briefly, *M. paratuberculosis* lysate and culture filtrate proteins were dialyzed to nanopure water by using 5k MWCO Spin-X® UF Concentrators (Corning, Corning, NY) and lyophilized overnight. The 20:80 CPTEG:CPH polymer (20 mg/mL) containing 2.5 wt% proteins was dissolved in methylene chloride. The solution was sonicated for 30 s to ensure uniform distribution of the protein throughout the solution. Particles were precipitated by pouring the solution into chilled pentane (1:250 methylene chloride:pentane) and collected via vacuum filtration. Nanoparticle size and morphology were characterized via scanning electron microscopy (FEI Quanta 250, FEI, Hillsboro, OR). The encapsulation efficiency was determined by incubating the nanoparticles in PBS at 37 °C. The released protein was quantified via a micro-bicinchoninic assay (Pierce) and compared with the amount of protein theoretically encapsulated. The final nanovaccine formulation was a combination of free protein and nanoparticle-encapsulated protein. A total of 1 mg of nanoparticles encapsulating 25 µg protein was suspended in 100 μL PBS containing 75 µg free protein per mouse. The mixture was sonicated for 30 s on ice to disperse any nanoparticle aggregates prior to administration.

### Mice vaccination and challenge

Female C57BL/6 mice (5–8 weeks of age) were obtained from Taconic Inc. and maintained in bio-safety level-2 containment. All procedures were in compliance with Institutional Animal Care and Use Committee, University of Wisconsin, Madison. Experiments were run in two trials (Table 2, *n* = 15/group) with trials I and II comprising of four and six vaccine groups, respectively. Animals were vaccinated subcutaneously as per the experimental design shown in Table 2. In all experiments, five mice from each group were sacrificed at 6 weeks post-vaccination. The remaining 10 animals in each group were challenged with 10^8^ CFU of *M. paratuberculosis* JTC-1285 in 100 μL of PBS, injected intraperitoneally (IP). The subcutaneous vaccination regime and intraperitoneal challenge model has been successfully employed before in mouse models and the resulting data translated well to ruminant systems such as in goats.^42,69,70^ The dose was confirmed by plating the serially diluted challenge inoculums on 7H10 plates. Mice were monitored daily for adverse reaction(s) from vaccination and for the progression of infection. At 6 weeks (Trial I), 12 weeks (Trials I and II) and 18 weeks (Trial II) post-challenge, mice (*n* = 5) were sacrificed and the liver, spleen, small intestine and mesenteric lymph nodes were harvested from each sacrificed mouse in order to quantify the bacterial burden. Organs were homogenized in 1 mL PBS and undiluted and 10-fold serial diluted samples were plated onto antibiotic free and selective media (hygromycin 30 μg/mL) to differentiate between *lipN* and challenge strain. When the selective media were not used, organs were plated onto standard 7H10 plates supplemented with ADC, mycobactin-J, and vancomycin (5 mg/mL), amphotericin B (30 mg/mL), and nalidixic acid (10 mg/mL) to reduce bacterial and fungal contamination. Finally, tissue sections were collected for histopathology and stained with hematoxylin and eosin.^41^ Slides were scored by a trained pathologist blinded to the samples. The animal experimental design is shown in Fig. 1.

**Table 2:** Vaccine groups used in Trial I and Trial II.

	Group	Dosage of protein
Trial I^a^	PAN-Lysate	75 µg soluble + 25 µg encapsulated
	Lysate (no PAN)	100 µg soluble
	Mycopar®	100 µL
	PBS	100 µL
Trial II^b^	PAN-Lysate	75 µg soluble + 25 µg encapsulated
	PAN-Cf	75 µg soluble + 25 µg encapsulated
	PAN-Cf + Lysate	75 µg soluble + 25 µg encapsulated
	LipN	10^8^ C.F.U.
	Mycopar®	100 μL
	PBS	100 μL

^a^Lysate obtained from *M. paratuberculosis* K10.

^b^Lysate obtained from *M. paratuberculosis* JTC-1285.

### Splenocyte isolation and stimulation

Spleens from five animals/group were aseptically harvested and placed in RPMI (Corning, Manassas, VA) supplemented with 1% FBS (Atlanta biological, Lawrenceville, GA), 1% L-glutamine (Gibco, Grand Island, NY), 1% penicillin-streptomycin (Mediatech, Inc. Manassas, VA) and 1% nonessential amino acids (Gibco). Spleens were pressed against the wire mesh screens to isolate splenocytes. The cells were washed with RPMI and resuspended in 1–2 mL of RBC lysis buffer (Tris buffered ammonium chloride) for 1 min, washed, and resuspended in RPMI with 10% FBS. Cells were counted using trypan blue dye to assess viability. A total of 10^6^ cells/well were seeded into 96-well round bottom plates and stimulated with 10 µg/mL of whole cell lysate of *M. paratuberculosis* K10 (Trial I) or *M. paratuberculosis* JTC-1285 (Trial II) and 100 U/mL IL-2 (BD Biosciences, San Jose, CA). A control of unstimulated cells from each sample was also plated and treated with 100 μL of media and 100 U/mL IL-2. Plates were incubated for 18 h at 37 °C, 5% CO_2_ followed by addition of Golgi-Plug to each well and incubated for an additional 5 h. Cells were harvested by centrifugation and stained with immune markers to be analyzed by flow cytometry and the supernatant was used to detect IFN-γ by ELISA.

### IFN-γ ELISA

Supernatant from the stimulated splenocytes was collected and tested for IFN-γ levels using Mouse IFN-γ ELISA MAX^TM^ Deluxe kit (Biolegend, San Diego, CA) following the manufacturer’s instructions. In brief, 96-well plates (Maxisorp-Immuno plates; Nunc) were coated overnight with capture antibody (monoclonal capture antibody specific for mouse IFN-γ) at 1:200 dilutions in the coating buffer at 4 °C. Plates were washed with PBST (137 mM NaCl, 2.7 mM KCl, 10.15 mM Na_2_HPO_4_, 1.76 mM KH_2_PO_4_, pH 7.4, 0.05% (v/v) Tween 20), blocked with 200 μL of assay diluent, and incubated on a shaking plate for 1 h at room temperature. Plates were washed five times with the PBST and 100 μL of sample and standards were added to the appropriate wells and incubated for 2 h on the shaking plate. Plates were washed with PBST and 100 μL of detection antibody (biotinylated rat monoclonal anti-mouse antibody) was added and incubated for 1 h at room temperature. After three more washes, 100 μL of Avidin-horse radish peroxidase conjugated solution was added to each well and incubated for 30 min on the shaking incubator. After a last few washes, 100 μL of freshly made 3’, 3, 5, 5’ -tetramethylbenzidine (TMB) substrate solution was added to the wells and incubated for 20 min in the dark. The reaction was stopped by adding 100 μL of stop solution (1 N H_2_SO_4_). The plates were read at a wavelength of 450 nm and analyzed with SoftMax Pro software (Molecular Devices, Sunnyvale, CA).

### Flow cytometry

Splenocytes from five individual mice per group were counted and 1 × 10^6^ cells/well were plated in 96-well plates. Stimulated cells were harvested by centrifuging at 400 × *g* for 10 min at 4 °C. Supernatants were removed and cells were washed with PBS twice. Fixable Viability Dye efluor 780 (eBioscience, San Diego, CA) was diluted in PBS (1/10) and added to each well except unstained control, incubated for 30 min in dark at 2–8 °C and CD16/Cd32 receptors were blocked with Fc block (BD Pharmingen, San Diego, CA). Cell surfaces were stained with cocktail of BUV496 conjugated anti-CD4 antibody, clone GK 1.5 (BD Pharmingen); BUV396 conjugated anti-CD8, clone 53-6.7 (BD Pharmingen); BV711 conjugated anti-CD25, clone PC61 (Biolegend) and incubated for 30 min in dark at 4 °C. Cells were washed with cold FACS buffer twice and resuspended in 200 μL of Fixation/Permeabilization working solution (Foxp3 staining buffer set, eBioscience, San Diego, CA). After 1 h incubation, cells were washed with 1X permeabilization buffer and stained intracellularly with APC conjugated anti-IFN-γ, clone XMG1.2 (BD Pharmingen); PE conjugated anti-IL-2 (BD Pharmingen); PEcy7 conjugated anti-TNFα, clone MP6-XT22 (eBioscience); and Alexa Fluor 780 conjugated anti-Foxp3, clone FJK16s (eBioscience). Cells were analyzed using BD FACSCalibur and data were analyzed using FlowJo software (FlowJo, LLC, Ashland, OR). Results were expressed as the increase in the percentage of the cells with positive staining relative to that of an unstimulated sample stained with the same antibody.

### Statistical analysis

Statistical analysis was performed using GraphPad Prism (La Jolla, CA). Data were analyzed using one-way ANOVA followed by Tukey’s multiple comparison. Results with *p* < 0.05 or better were considered significant. All research reported here was conducted in accordance with all relevant guidelines and procedures, and that the work was approved by the University of Wisconsin–Madison.

### Reporting summary

Further information on research design is available in the Nature Research Reporting Summary linked to this article.

## Supplementary information

Supplemental Information

Reporting Summary

## Data Availability

All data presented in this paper are available through this report or the accompanied supplemental tables and figures.
