# Supplementary material for: A single dose polyanhydride-based nanovaccine against paratuberculosis infection
Source: NPJ Vaccines. 2020 Feb 14;5:15. doi: 10.1038/s41541-020-0164-y (PMC7021715; doi:10.1038/s41541-020-0164-y)
Supplement: Supplementary file 1 — Supplemental Information [file 41541_2020_164_MOESM1_ESM.pdf]

Supplemental figures and tables

A Single Dose Polyanhydride-based Nanovaccine Against Paratuberculosis Infection.

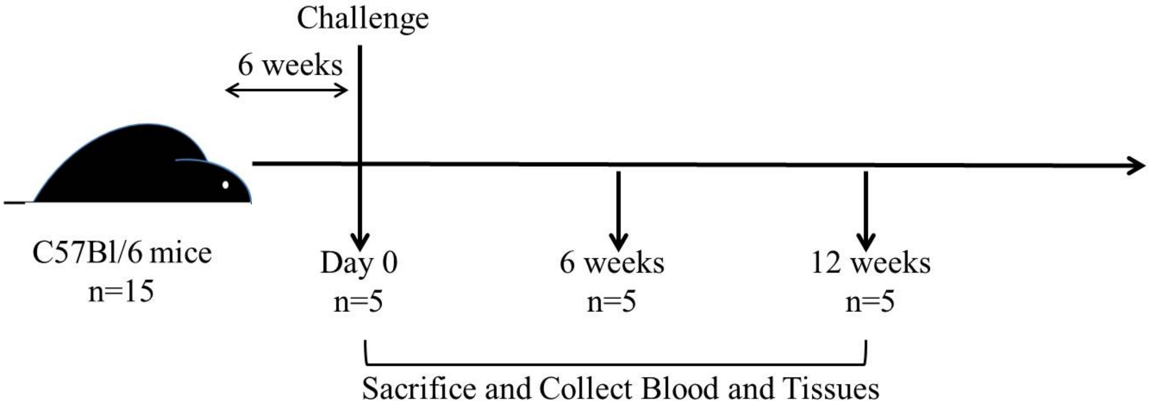

Supplemental Fig. 1. Experimental design. Five- to eight-week old female C57BL/6 mice were vaccinated (vaccine groups of Trial I) with subcutaneous injection and challenged 6 weeks later with virulent strain of *M. paratuberculosis* JTC-1285 by intraperitoneal route. Mice (n = 5) were sacrificed at 6 and 12 weeks post-challenge. Tissues were collected to measure bacterial burden and IFN-γ.

a)

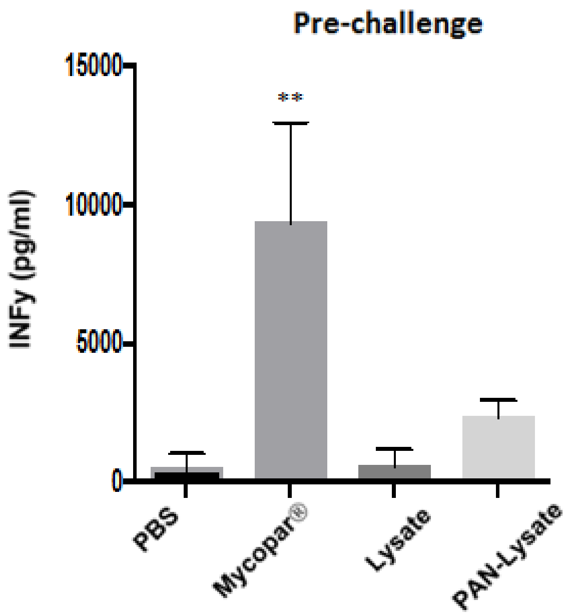
Pre-challenge
15000
10000
5000
0
INFγ (pg/ml)
**
PBS
Mycopar®
Lysate
PAN-Lysate

b)

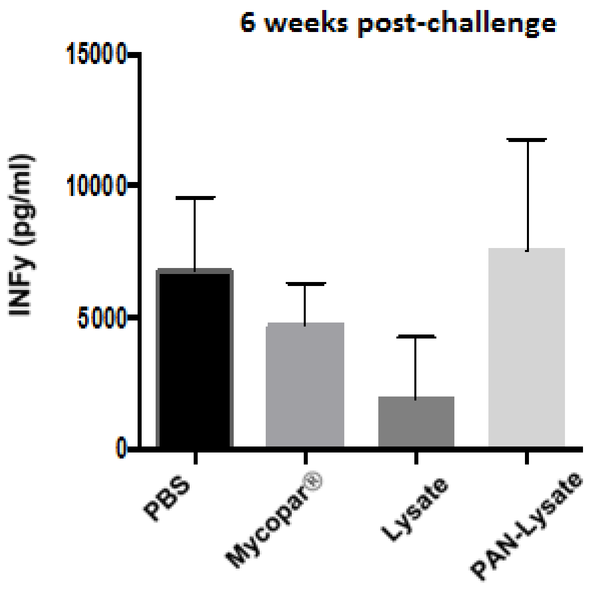
6 weeks post-challenge
15000
10000
5000
0
INFγ (pg/ml)
PBS
Mycopar®
Lysate
PAN-Lysate

c)

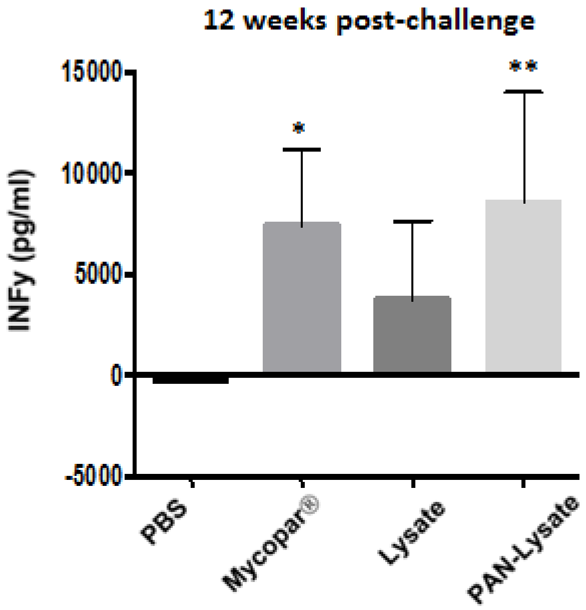

Supplemental Fig. 2. Antigen-specific T cell immune response. C57BL/6 mice were immunized with Trial I vaccine groups. At 6 weeks post-immunization five mice from each group were euthanized and rest of them were challenged with M. paratuberculosis strain, JT-1285. Lymphocytes were isolated from the spleen and stimulated with the M. paratuberculosis lysate and IFN-γ was measured in the culture supernatants by the capture ELISA at pre-challenge time point (a) and 6 weeks post-challenge (b) and 12 weeks post-challenge (c).

a)

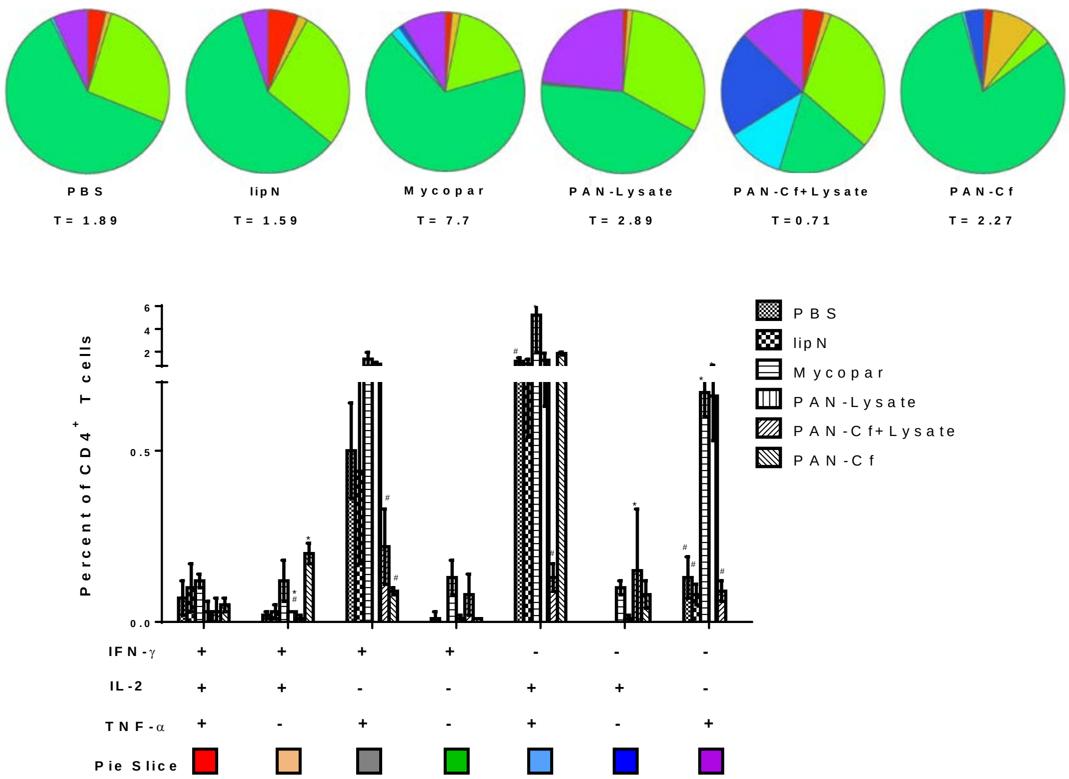
PBS
T= 1.89
lipN
T= 1.59
Mycopar
T= 7.7
PAN-Lysate
T= 2.89
PAN-Cf+Lysate
T=0.71
PAN-Cf
T= 2.27
Percent of CD4+ T cells
PBS
lipN
Mycopar
PAN-Lysate
PAN-Cf+Lysate
PAN-Cf
IFN-γ
IL-2
TNF-α
Pie Slice

b)

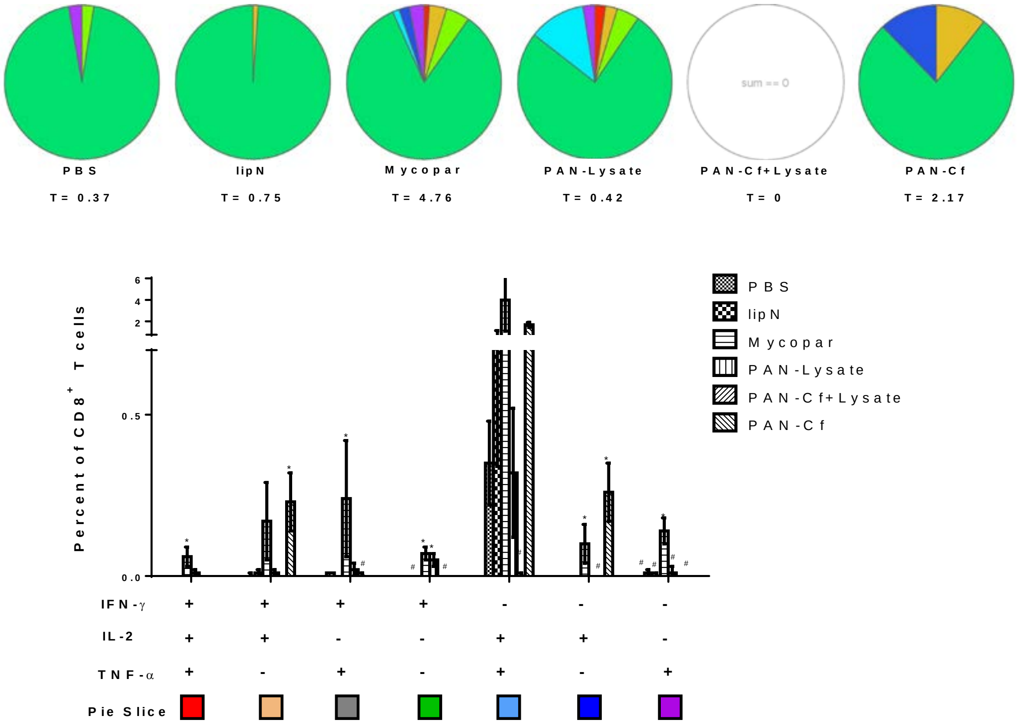
sum == 0
PBS
T= 0.37
lipN
T= 0.75
Mycopar
T= 4.76
PAN-Lysate
T= 0.42
PAN-Cf+Lysate
T= 0
PAN-Cf
T= 2.17
Percent of CD8+ T cells
PBS
lipN
Mycopar
PAN-Lysate
PAN-Cf+Lysate
PAN-Cf
IFN-γ
IL-2
TNF-α
Pie Slice

Supplemental Fig 3 . Late cellular responses in vaccine groups following challenge with a wild type strain of *M. paratuberculosis*. At 18 WPC, mice (N=5) from each vaccinated group were euthanized and their spleens were harvested to isolate lymphocytes. The lymphocytes were isolated from the spleens and stimulated with whole cell lysate of *M. paratuberculosis* for 24 h. Cells were then stained for $CD4^+$(a) and $CD8^+$ (b) cell surface markers and intracellular cytokines and were measured by flow cytometry. The total percentage of T cells secreting particular cytokines are indicated below each pie chart (denoted by T= number). The error bars show the standard error of the mean for five individually analyzed mice. * indicates $p < 0.05$; ** indicates $p < 0.001$. * denotes comparison with PBS while # denotes comparison with Mycopar®. Results were expressed as the increase in the percentage of the cells with positive staining relative to that of an unstimulated sample stained with the same antibody.

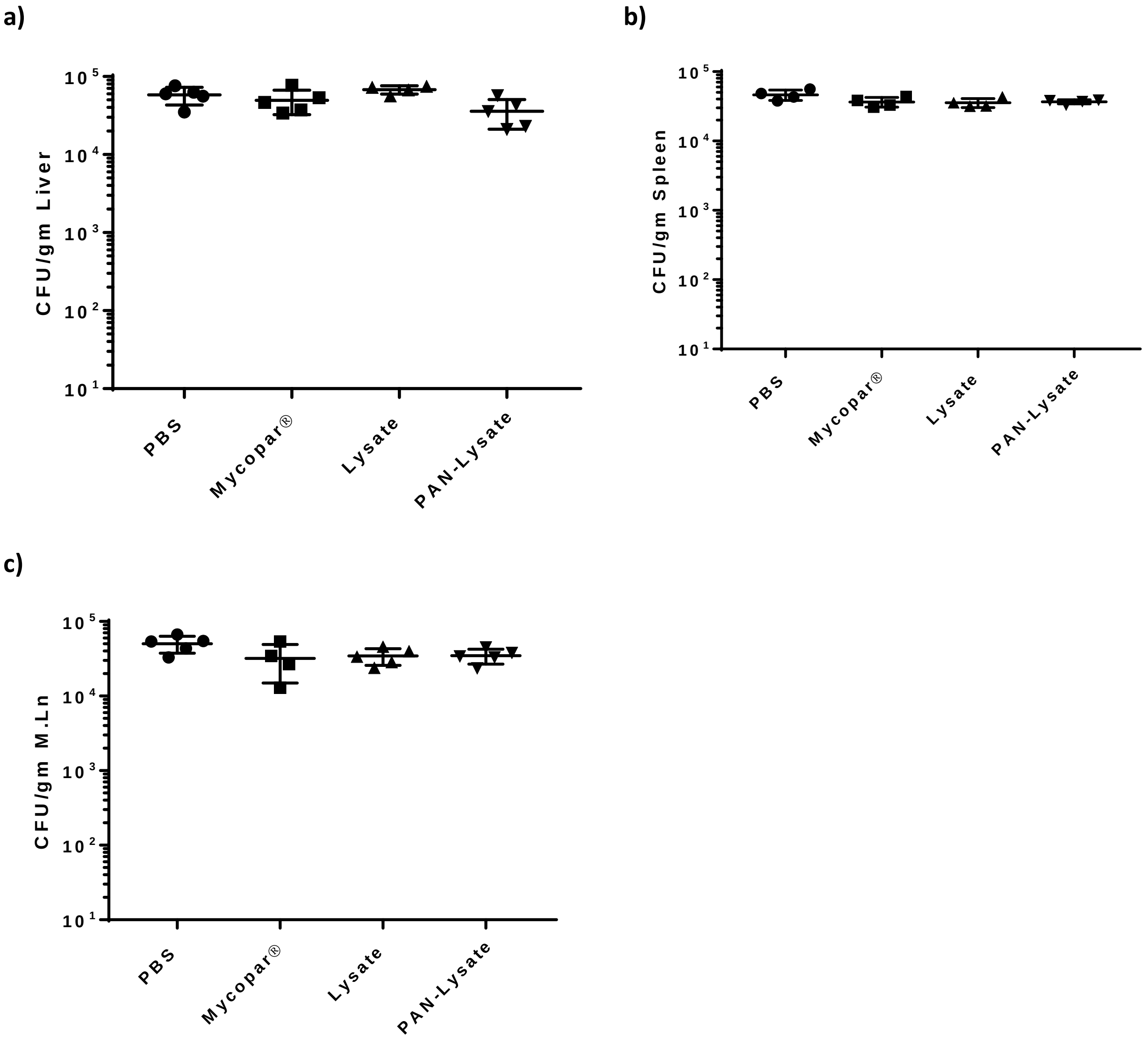

Supplemental Fig. 4. Bacterial load at 6 weeks post-challenge. In Trial I**,** 6 weeks following *M. paratuberculosis* challenge, liver, spleen and mesenteric lymph nodes were harvested to measure bacterial load. Shown are the total counts for each individual animal in spleen (a), liver (b) and mesenteric lymph node (c). Error bars indicate standard deviation.

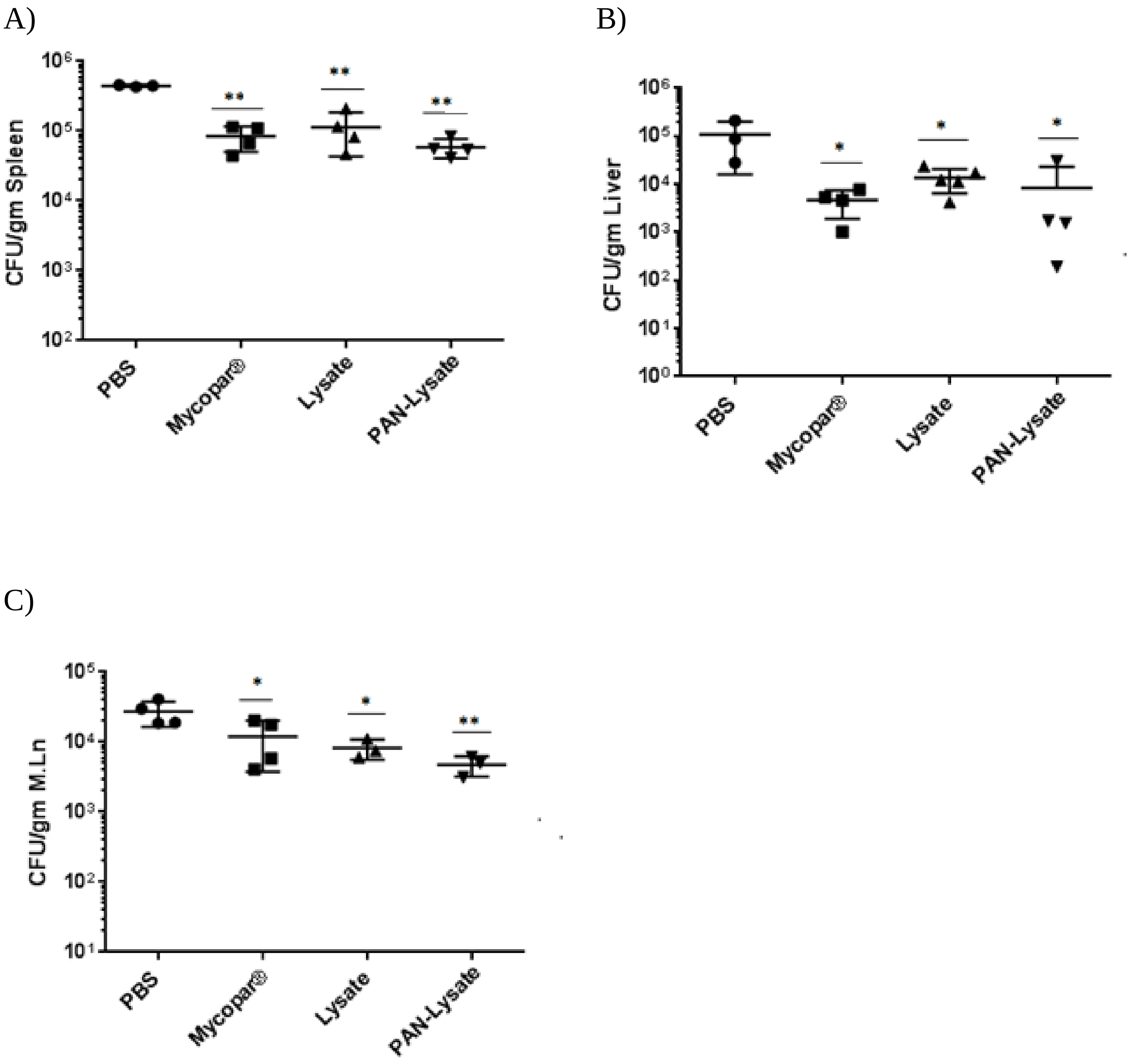

Supplemental Fig. 5. Bacterial load at 12 weeks post-challenge. In Trial I**,** 12 weeks following *M. paratuberculosis* challenge, liver, spleen and mesenteric lymph nodes were harvested to measure bacterial load. Shown are the total counts for each individual animal in Liver (a), Spleen (b) and mesenteric lymph node (c). Error bars indicate standard deviation *, $p<0.05$; **, $p<0.001$.

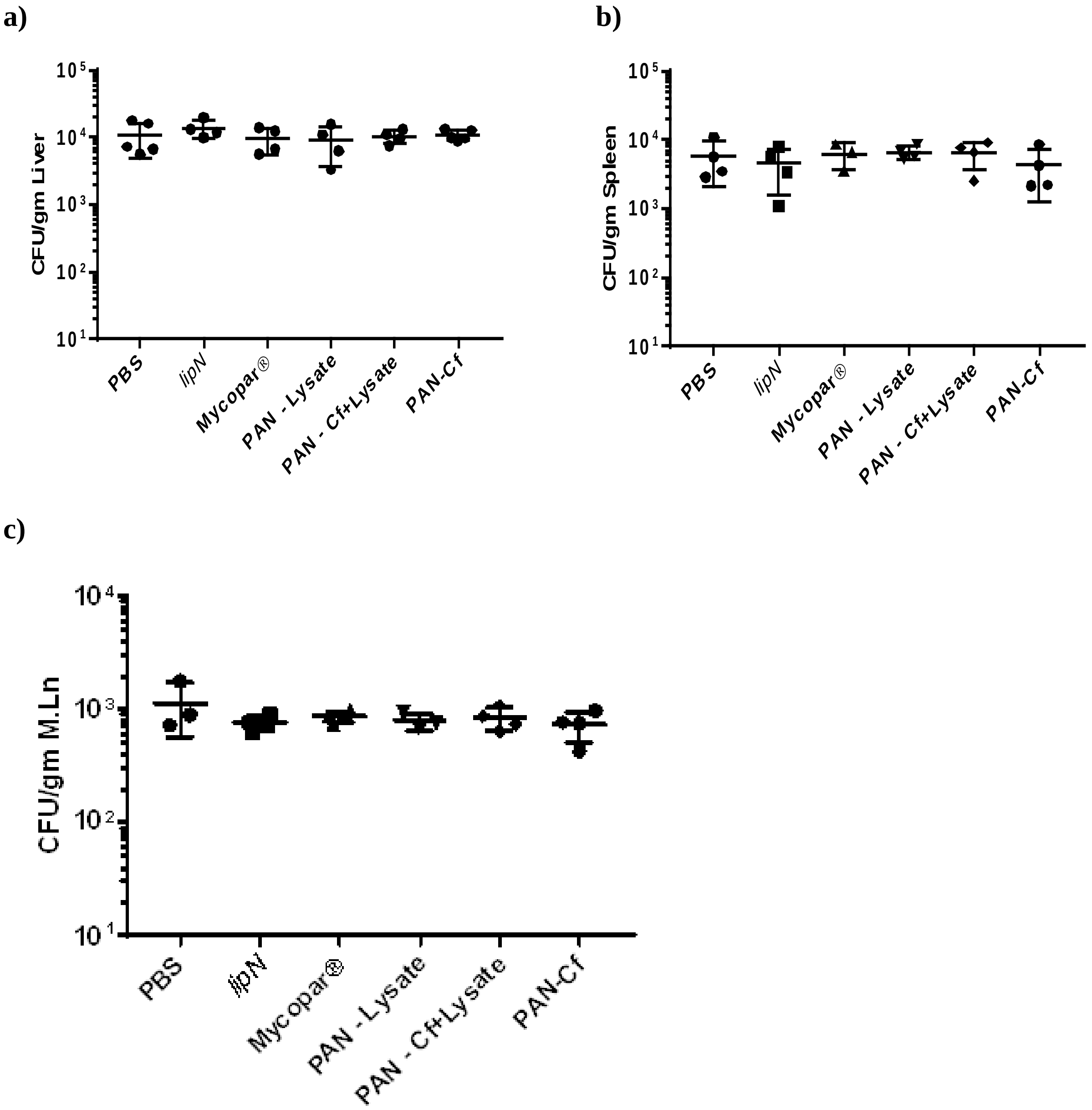

Supplemental Fig. 6. In Trial II**,** liver, spleen and mesenteric lymph nodes were harvested to measure bacterial load18 weeks following *M. paratuberculosis* challenge. Shown are the total counts for each individual animal in Liver (a), Spleen (b) and mesenteric lymph node (c). Error bars indicate standard deviation. No significance was observed among treatment groups.

and stimulated with whole cell lysate of *M. paratuberculosis* for 24 h. Cells were then stained for $CD4^+$(A) and $CD8^+$ (B) cell surface markers and intracellular cytokines and were measured by flow cytometry. The total percentage of T cells secreting particular cytokines are indicated below each pie chart (denoted by T= number). The error bars show the standard error of the mean for five individually analyzed mice. * indicates $p < 0.05$; ** indicates $p < 0.001$. * denotes comparison with PBS while # denotes comparison with Mycopar®.

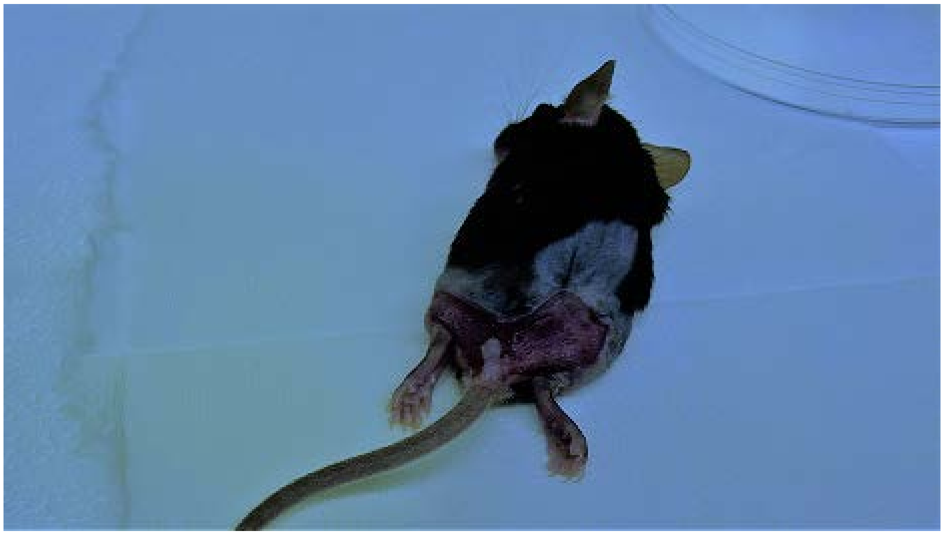

Supplemental Fig 7. This picture shows a lesion at the site of Mycopar vaccine administration in a mouse which progressed over time and led to euthanasia. Four out of fifteen mice exhibited such lesions.

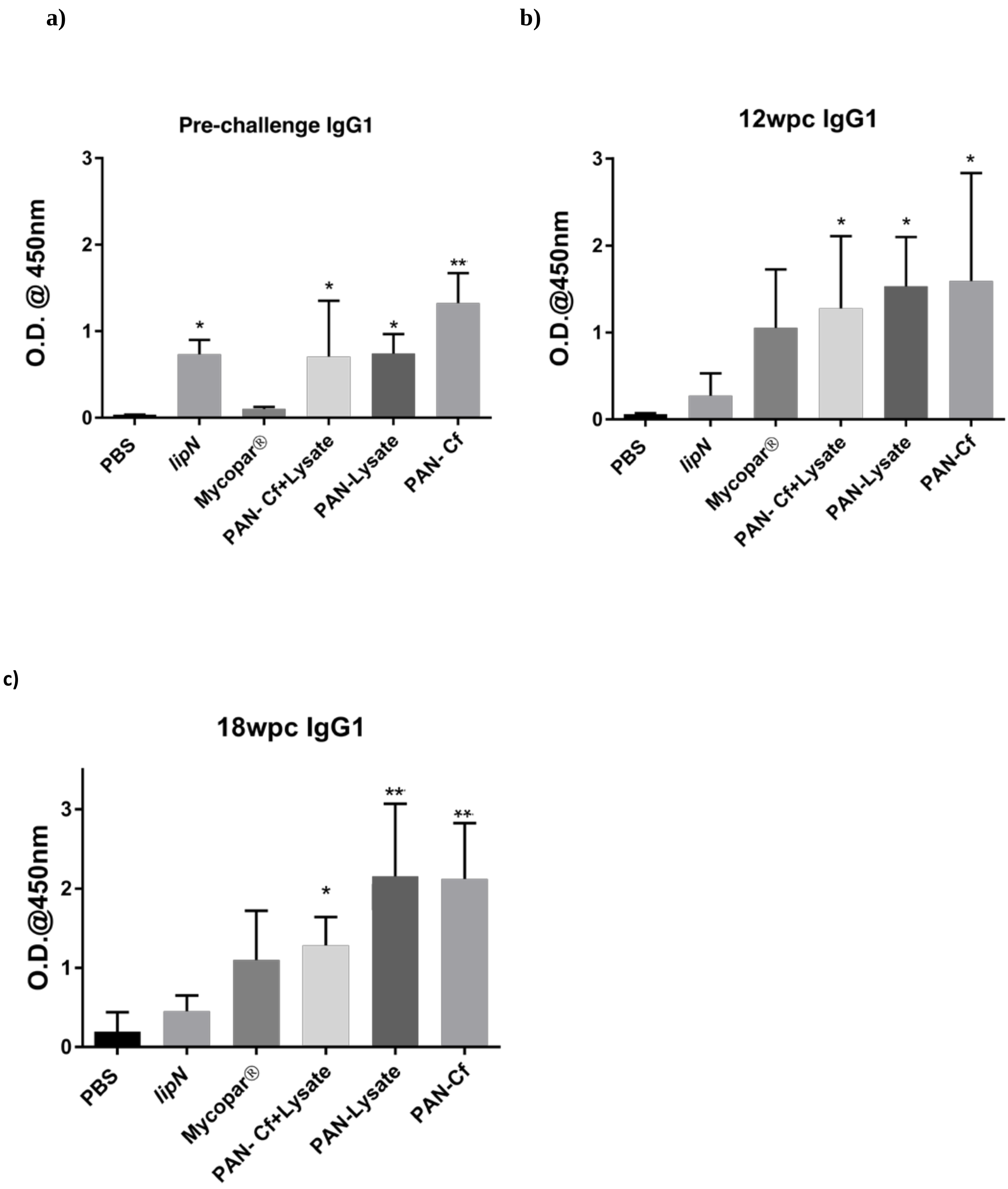

Supplemental Fig 8. In Trial II, levels of IgG1 antibody response (displayed as optical density, O.D.) to the whole cell lysate of *M. paratuberculosis,* were measured in the sera of immunized

mice at pre-challenge, 12 WPC and 18 WPC. Sera were diluted to 1:100 and ELISA was performed as described in Material and Methods. Data are representative of five animals in each group at all the time points. Error bars show the standard error of the mean. $p<0.05$ was considered statistically significant. *, $p<0.05$; **, $p<0.001$.

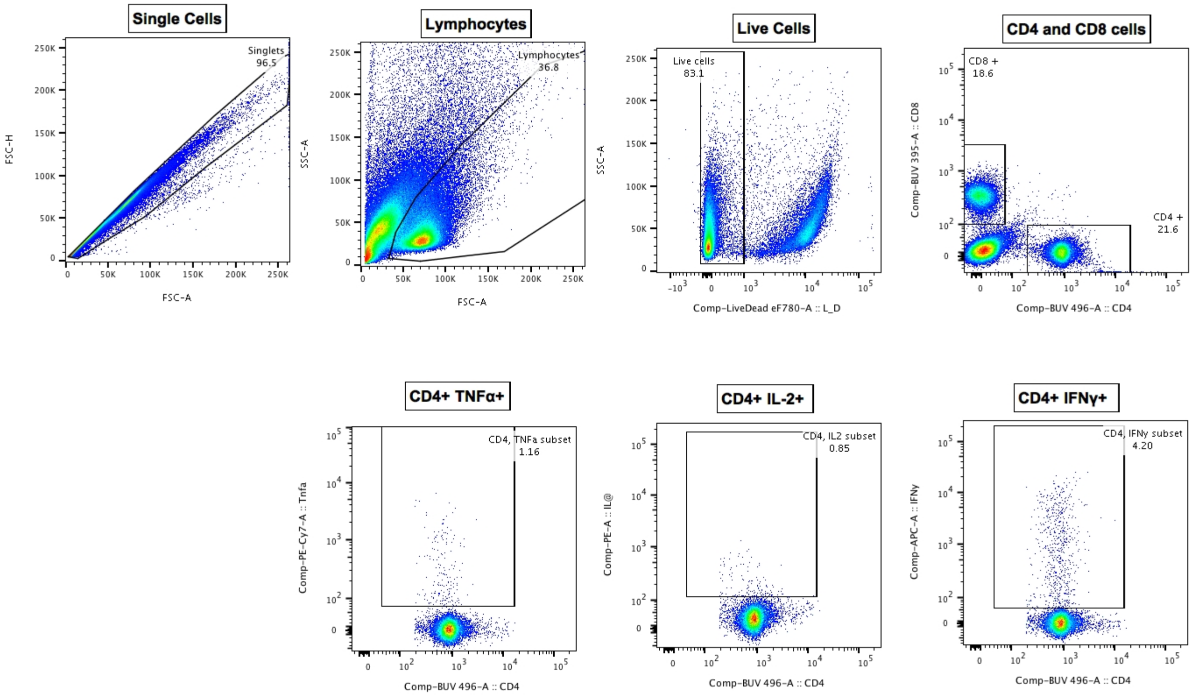

Supplemental Fig 9. Gating strategy to identify cytokine secreting $CD4^+$ and $CD8^+$ T cell population.

Supplemental Table 1. Pre-Challenge liver histopathology at 6 WPV.

Granulomatous inflammation (GI) in Liver

Vaccine groups	Severity score*	
	Animal 1	Animal 2
PBS	N	N
Mycopar	3	1
PAN - Lysate	2	1
PAN - Cf +Lysate	N	N
PAN - Cf	N	1
lipN	N	1

*Severity index of granulomatous lesions is as follows; N = normal, 1 = minimal, 2 = mild, 3 = moderate, 4 = severe, 5 = massive.
